# Health-related parental indicators and their association with healthy weight and overweight/obese children’s physical activity

**DOI:** 10.1186/s12889-018-5582-7

**Published:** 2018-05-31

**Authors:** E. Sigmund, D. Sigmundová, P. Badura, A. Madarasová Gecková

**Affiliations:** 1 Institute of Active Lifestyle, Faculty of Physical Culture, Palacký University Olomouc, Tr. Miru 117, 77111 Olomouc, Czech Republic; 2 Department of Health Psychology, Faculty of Physical Culture, Safarik University, Košice, Slovakia; 3 Graduate School, Košice Institute for Society and Health, Safarik University, Košice, Slovakia

**Keywords:** Step counts, Organized leisure time physical activity, Preschool and school-aged children, Overweight and obesity

## Abstract

**Background:**

Although it is accepted that parents play a key role in forming children’s health behaviours, differences in parent-child physical activity (PA) have not previously been analysed simultaneously in random samples of families with non-overweight and overweight to obese preschool and school-aged children. This study answers the question which of the health-related parental indicators (daily step count (SC), screen time (ST), and weight status and participation in organized leisure-time PA) help their children achieve the step count recommendations.

**Methods:**

A nationally representative sample comprising 834 families including 1564 parent-child dyads who wore the Yamax Digiwalker SW-200 pedometer for at least 8 h a day on at least four weekdays and both weekend days and completed a family log book (anthropometric parameters, SC, and ST). Logistic regression analyses were used to investigate whether parental achievement of the daily SC recommendation (10,000 SC/day), non-excessive ST (< 2 h/day), weight status, and active participation in organized PA were associated with children’s achievement of their daily SC (11,500 SC/day for pre-schoolers and 13,000/11,000 SC/day for school-aged boys/girls).

**Results:**

While living in a family with non-overweight parents helps children achieve the daily SC recommendation (mothers in the model: OR = 3.50, 95% CI = 2.29–5.34, *p* < 0.001; fathers in the model: OR = 2.41, 95% CI = 1.37–4.26, *p* < 0.01) regardless of their age category, gender, or ST, for families with overweight/obese children, only the mother’s achievement of the SC recommendations and non-excessive ST significantly (*p* < 0.05) increase the odds of their children reaching the daily SC recommendation. The active participation of children in organized leisure-time PA increases the odds of all children achieving the daily SC recommendations (OR = 1.80–2.85); however, for overweight/obese children this remains non-significant. The participation of parents in organized leisure-time PA does not have a significant relationship to the odds of their overweight/obese or non-overweight children achieving the daily SC recommendations.

**Conclusions:**

The mother’s health-related behaviours (PA and ST) significantly affect the level of PA of overweight/obese preschool and school-aged children. PA enhancement programmes for overweight/obese children cannot rely solely on the active participation of children in organized leisure-time PA; they also need to take other family-based PA, especially at weekends, into account.

## Background

According to many theoretical models and theories [1–4] describing human behaviour, the direct influence of parents on the social development and behaviour of their children is firmly established. Parents have been termed the primary gatekeepers of their children’s health [5]. The parent-child physical activity (PA) or screen time relationship has been studied in a wide range of social [6], psychological [7, 8], educational, and health-related disciplines [9, 10], with an emphasis on healthy child development [6–10].

A focus on parent-child dyad analysis in preschool and preadolescent children is key to understanding the factors that are essential for shaping the active lifestyle of children, which persists until adulthood [11, 12]. Numerous longitudinal studies confirm the persistence of obesity arising in preschool age to adolescence [13, 14] and adulthood [15, 16]. An active lifestyle throughout childhood and adolescence could thus prevent the development of obesity in young adulthood [13]. Additionally, the relationship of parent-child overweight/obesity has already been proven to exist at preschool level [17–19]. Importantly, the risk of the transmission of obesity from childhood to adulthood seems to be stronger when a child has one obese parent than in obese children without obese parents [16].

Objective parent-child PA and screen time measurements have been analysed globally – Western Europe [7, 20–22] and Northern Europe [23], North and South America [17, 24–29], Africa and South Asia [17], and Australia and New Zealand [30, 31]. The emphasis in such studies is placed, for instance, on active parental participation in organized or non-organized PA [24, 26, 29, 31], parental support for PA [7, 8, 32, 33] and parenting styles [25, 32]. Other studies investigate role of parental rules and restrictions concerning screen time [30], parental education and family socioeconomic status [7, 17, 20, 21, 23, 30], variation in the weekday-weekend PA and screen time relationship [7, 24, 26, 27, 29, 33], and level of body weight [17, 20, 22, 27, 29, 34]. However, similarly valuable studies of objective parent-child PA and screen time measurements in the countries of Central and Eastern Europe are lacking.

Except for selected meta-analyses and reviews [35, 36], other parent-child PA/screen time studies are focused separately either on preschoolers [20, 22, 30, 31] or school-aged children and adolescents [7, 8, 17, 21, 24, 25, 27, 29, 32–34]. The parent-child PA/screen time relationship in a broader age spectrum of preschool and school-aged children is seldom analysed [26, 37]. In addition, parent-child PA/screen time relationships have been analysed only rarely in the countries of Central, Southern, and Eastern Europe [38, 39]. The countries of Central, Southern, and Eastern Europe, however, belong among those European countries that are challenged by the trend of an increase in childhood overweight/obesity [40, 41], screen time behaviours [42], and reduced PA [43]. Public health-related disciplines in these countries need to possess relevant information on the roles of families in shaping the active lifestyle of their children. It is because these countries tend to repeat the behavioural health-related patterns of children previously witnessed in children and adolescents from Western high-income countries, e.g. a decrease in PA, an increase in sedentary behaviours (especially screen time activities), an increase in the excessive consumption of sweetened beverages, and a greater intake of fast food [44]. Such behaviours consequently lead to increased rates of overweight and obesity [45–47]. Previous parent-child study [48] also pointed out that active participation of parent/children in organized PA as a promising “vehicle” to promote active lifestyle in children. However, it is not known whether active participation in leisure-time organized PA helps both healthy weight and overweight/obese children to reach PA recommendations. The selected physical activity behaviours are more easily modifiable, thus influenceable through eventual intervention programs or stimuli, than socioeconomic status, structure, or place of residence, which play their role too.

The study attempts to bridge the research gap of insufficient relevant information in Central European nations concerning the parent-child PA/screen time relationship in a random sample of Czech families with preschool and school-aged children whose body weight ranges from normal to overweight or obese with regard to the parents’ body weight and their participation in organized leisure-time PA.

This study aimed to estimate which of the health-related parental indicators (daily step counts, amount of screen-based entertainment time, weight status, and participation in organized leisure-time PA) help their preschool and school-aged children achieve their step count recommendations. Furthermore, we assessed whether the associations differ by gender of parents, weight status and participation in organized PA of children.

## Methods

This research involved the use of data collected from the Czech-based Parent Child PA Care (PACPAC) Study. PACPAC is a three-cohort study that investigates the parent-child PA/screen time relationship in families with pre-schoolers (aged 3–6.49 years) and school-aged children (aged 6.5–12 years). This study collected data on parent-child dyads in the spring (from March until June) and autumn (from September until November) months between 2013 and 2016. The Ethical Committee of the Faculty of Physical Culture, Palacký University Olomouc approved the study design and protocol for families with school-aged children (ref. no. 17/2013) on 25 March 2013 and for families with preschool children (ref. no. 57/2014) on 10 December 2014.

### Sample and inclusion criteria

Participants were recruited by means of two-stage stratified random sampling. In the first stage, nine out of 14 administrative regions, three of each in the lowest, middle, and highest terciles for gross domestic product in the Czech Republic, were randomly selected. In the second stage of sampling, the selection of kindergarten and primary schools respected the distribution of the urban-rural population in the Czech Republic [49]. A total of 296 families with preschool children and 1610 families with school-aged children were addressed in writing with an invitation to participate in the study before a joint meeting with the authors of the study. Participating children and their parents were predominantly white Caucasian (> 98%), which is representative of the ethnic demographics of the Czech Republic [50].

The disproportion of the sample in terms of age, i.e. higher number of school-aged children, was due to wider age range of school-aged children investigated compared with pre-schoolers. Furthermore, while school attendance is compulsory, kindergarten attendance (except for the last pre-school year is optional). The objectives, procedures/measures, and course of the project were thoroughly explained to the invited parents of the children and teachers and school/kindergarten employees at a joint meeting in each of the schools/kindergartens that participated. Written consent to participation in the study was obtained from 223 families with pre-schoolers (a response rate of 75%) and 1112 families with school-aged children (a response rate of 69%) at the end of the joint meeting (Table 1). The data of 38 families with preschool children and 463 families with school-aged children was not included in the analyses because of incompleteness (missing data on body weight, height, or age or an incomplete record of PA/screen time data in the family log book) or invalidity (an absence of more than 1 day of the child from kindergarten/school or insufficient (< 8 h) time spent wearing the pedometer each day). In accordance with the recommendations of previous studies [51, 52], the final analyses included only data from parent-child pairs (mother and child *n* = 707 and father and child *n* = 455) of participants who wore the pedometer for at least 8 h a day on at least four weekdays and both weekend days (Table 1).

**Table 1:** Summary sample characteristics (*N*, %, mean (standard deviation))

	Families with preschool children		Families with school-aged children	
Respondents addressed to participate	296 (100%)		1610 (100%)	
Written consent obtained (%*)	223 (75.3%)		1112 (69.1%)	
Initiating research (%*)	215 (72.6%)		1040 (64.6%)	
The final set with valid data (%*)	185 (62.5%)		649 (40.3%)	
			1st-3rd grade	4th–5th grade
Parent-child dyads		M (SD)	M (SD)	M (SD)
Mothers	*N*	164	289	254
	Age (years)	36.19 (4.20)	38.19 (4.04)	38.94 (4.05)
	BMI (kg/m^2^)	24.02 (3.99)	23.42 (3.68)	23.91 (3.82)
	Overweight	24.39%	19.04%	20.87%
	Obese	9.76%	8.30%	7.87%
Fathers	*N*	107	187	161
	Age (years)	38.91 (5.29)	40.15 (4.25)	41.38 (5.22)
	BMI (kg/m^2^)	26.04 (3.34)	26.81 (3.42)	26.64 (3.19)
	Overweight	50.47%	49.20%	63.31%
	Obese	11.21%	17.11%	14.91%
Girls	*N*	88	173	159
	Age (years)	5.59 (0.74)	7.92 (0.81)	10.61 (0.74)
	BMI (kg/m^2^)	15.13 (2.41)	16.38 (2.51)	17.70 (3.00)
	Overweight	9.09%	15.60%	13.83%
	Obese	9.09%	6.94%	7.55%
Boys	*N*	97	172	145
	Age (years)	5.68 (0.73)	8.00 (0.84)	10.62 (0.75)
	BMI (kg/m^2^)	15.41 (1.81)	16.65 (2.86)	17.67 (2.75)
	Overweight	6.18%	15.12%	17.93%
	Obese	9.28%	13.95%	8.28%

%* – percent of the initial sample addressed

% – overweight/obesity; overweight or obesity in children represents a BMI from the 85th to 97th or greater than the 97th percentile of the WHO growth charts [71, 72]. Overweight and obesity in parents represents a BMI from 25 kg/m^2^ to 29.9 kg/m^2^ and greater than or equal to 30 kg/m^2^, respectively [73]

*N* number, *M* arithmetic mean, *SD* standard deviation, *BMI* Body mass index

### Procedures and measures

During the baseline joint meeting the invited parents and kindergarten/school teachers were thoroughly acquainted with the procedures and course of the monitoring of PA and recording of the step count/screen time -related data into a family log book. The parents received instructions regarding how to use a pedometer and the process of recording the monitored values in the family log book. The family log book is composed of three sections – the first to record the anthropometric parameters of all the family members, the second for the PA-related data (step count, participation in organized PA), and the third for recording the screen time (type and duration) activities [53].

The parents were asked to record the demographic and anthropometric parameters (birth date of children and age of parents, gender, body height (with 0.5-cm accuracy), and weight (with 0.5-kg accuracy)) of all the participating family members in the first section of the family log book before the start of the one-week monitoring of PA/screen time behaviour. The parents were instructed how to measure their own body height and weight at home, as well as the height and weight of their children. The parental home measurement of the body weight and body height of their preschool [54] and school-aged children and adolescents [55] are sufficiently valid tools for determining the Body Mass Index (BMI) for the subsequent identification of overweight and obesity in children [55, 56].

The PA of all the participants in the three-cohort study was monitored using the same type of unsealed Yamax Digiwalker SW-200 pedometer (Yamax Corporation, Tokyo, Japan). Participants were instructed to wear the pedometer on their right hip for eight consecutive days during waking hours except when bathing, showering, and dressing. Every morning after their personal hygiene, the parents reset the pedometers, attached them to the right hip (their children’s and their own), and recorded the time of the resetting in the family log book. In the evening, the parents removed the pedometers and, together with their children, recorded the time and overall daily step count of all the participating family members in the log book. In addition, parents also recorded whether they or their children actively participated in organized leisure-time PA during the day into the log book. Organized leisure-time PA covers all kinds of structured intentional PA performed under the guidance of an educator (such as teacher, coach, and instructor) and does not include lessons of physical education during school/kindergarten time [53]. Those who participated at least once a week, were considered to be participants in organized leisure-time PA. The values from the first day of monitoring were not included in the final analyses because of insufficient time spent wearing the pedometer and because of the novelty of wearing it, which could have affected the level of the participants’ PA [51]. The pedometer-based monitoring of ambulatory PA is an objective, cheap, and unobtrusive method providing a reasonable assessment of a child’s day-long PA, albeit only when the total amount of PA, not its intensity, is of interest [57, 58]. The good validity and reliability of the hip-worn Yamax Digiwalker SW-200 step count measurement support the use of the Digiwalker for assessing free-living PA in preschool [58] and school-aged children [57–59], as well as in adults [60].

The sedentary behaviour of all family members was self-reported in the family log book by the parents. However, rather than all types of sedentary behaviours, attention was focused on screen time activities only, since they allow more accurate discrimination of health-risk behaviours than total sedentary behaviour does [61, 62]. The duration and type of entertainment screen time (sitting/lying while watching TV and sitting/lying in front of a PC (notebook, tablet, or smartphone) and not for school/work purposes) was recorded with an accuracy of 10 min by the parents, together with their children, each evening. The parent-proxy assessment of the amount of time their children spent watching TV daily exhibits an acceptable 7-to-14-day test-retest reliability (ICC = 0.78, *p* < 0.001) [63] and shows a strong positive correlation with direct home time-lapse videos (*r* = 0.84, *p* < 0.001) [64].

### Data management

The step count/screen time data was reviewed to check for extreme values. The daily step count variable represented the mean difference between the morning (pedometer turned on) and evening (pedometer turned off) step count/screen time on the days of the week that were monitored. Daily step count values below 1000 or exceeding 30,000 were truncated to these recommended limit values, respectively [37, 51], and included in the analyses. Weekly averages were calculated by adding 2/7 of the weekend day average and 5/7 of the weekday average. If step count and screen time were recorded on four weekdays, data for the one missing weekday based on the participant’s personal mean scores was added. The participants whose step count/screen time data was missing for more than 1 day were excluded from the analyses. The daily step count recommendation for preschool children was set at a value of 11,500 steps/day [65]. For school-aged children, a value of 13,000 steps/day was applied for boys and 11,000 steps/day for girls [53, 66], and for adults it was a value of 10,000 steps/day [67]. Daily screen time shorter than 10 min was not counted and if it was longer than 14 h it was shortened to this recommended value [53]. Excessive screen time for preschool children was defined as more than 1 h/day [68, 69] and for school-aged children [61, 62] and for adults as two or more hours a day [70].

The BMI was calculated as the body weight (kg) divided by the square of body height (m). The chronological age of all family members was calculated from their date of birth until the first monitoring day. Age-specific cut-off points [71–73] were used to define the prevalence of overweight/obesity. Overweight or obesity in children is represented by a BMI from the 85th to 97th or greater than the 97th percentile of the WHO growth charts, respectively [71, 72]. Overweight and obesity in parents is represented by a BMI from 25 kg/m^2^ to 29.9 kg/m^2^ and greater than or equal to 30 kg/m^2^, respectively [73].

### Statistical analyses

**Fig. 1:**
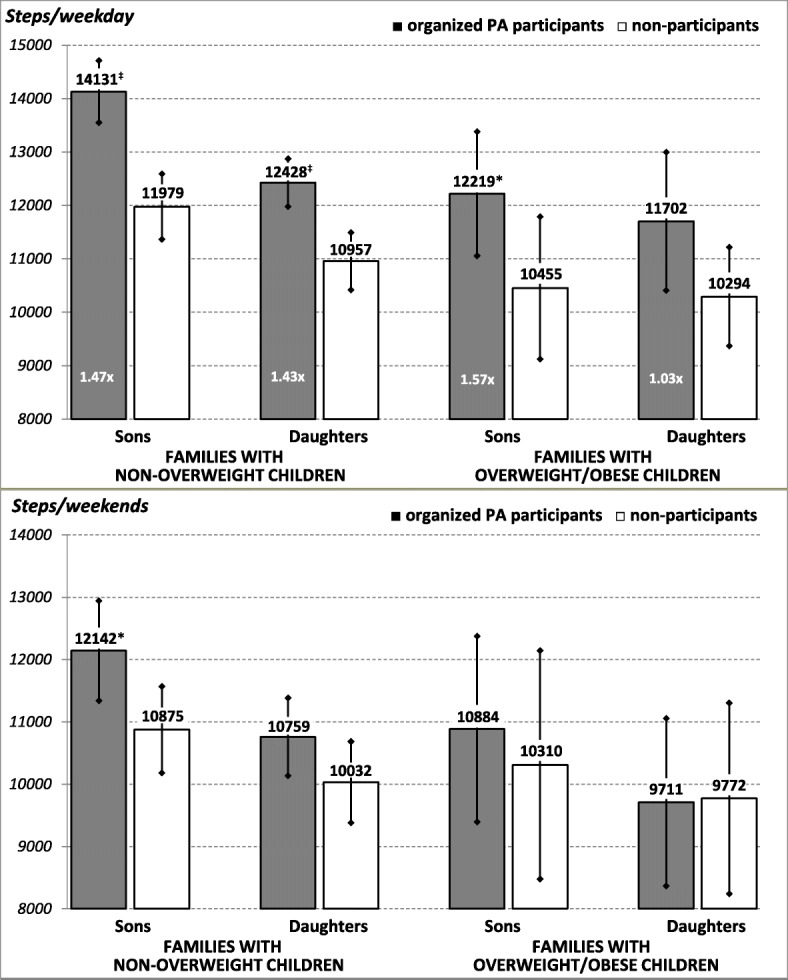
Comparison of children’s daily step counts (mean and 95% CI) on weekdays and at weekends. Legend: CI – confidence interval; x – mean number of sessions of organized leisure-time PA per week. The statistical significance of the differences between participants in organized PA and non-participants in terms of their daily step count (independent t-test (2-tailed)) is expressed as *p < 0.05 and ^‡^*p* < 0.001

Descriptive characteristics for the daily step count, prevalence of overweight and obesity, percentages of participants who met the daily step count recommendations, percentages of participants with excessive daily screen time, and frequency of participation in organized leisure-time PA were calculated for all family members (girls, boys, mothers, and fathers) separately. Summary sample characteristics are represented by means and standard deviations. The daily step count data is presented in the form of means and a 95% confidence interval or percentages. Logistic regression models (Enter Method) were used to identify which family-related variables (achievement of the recommended daily step count, excessive screen time, parental overweight/obesity, participation in organized leisure-time PA, and the gender of children) were associated with children of normal body weight and overweight/obese children achieving the step count recommendations separately). The models were adjusted for age category and gender of children. We used ordinary single-level regression, because initial analyses were not significantly altered by clustering of data by school/kindergarten. An independent t-test (2-tailed) was used to compare the daily step count (as presented in Fig. 1) of participants and non-participants in organized leisure-time PA split by gender and the level of body weight of the children. The Statistical Package for the Social Sciences (SPSS) for Windows v.22 software (IBM Corp. Released 2013. Armonk, NY, USA) was used for data management and all statistical analyses. The alpha level of significance was set at the minimum value of 0.05 for all the statistical analyses.

## Results

Among the children (420 girls and 414 boys), the prevalence of overweight was observed in 13.6% (7.6% were classified as obese) of the girls and 14.0% (10.9% were classified as obese) of the boys. Of the 185 preschoolers, the incidence of overweight was detected in 7.6% (and that of obesity in 9.2%) of them, while among the school-aged children the representation of overweight amounted to 15.6% (and that of obesity to 9.3%) out of the total number of 649 school-aged children (Table 1).

The relationship between children’s PA and parental indicators of health-related behaviours is presented in Table 2. Using the binary measures of achieving the recommended levels of daily step count, we found strong positive associations between mothers’ and children’s step count (*p* < 0.05), regardless of the maternal and children’s level of body weight. Fathers’ PA and level of body weight were only significantly associated with non-overweight children achieving the daily step count recommendation (Table 2).

**Table 2:** Logistic regression analysis: odds ratios and 95% confidence intervals for meeting the daily step count recommendations in non-overweight and overweight/obese children, separately for the mother-child and father-child pairs included in the model

	Meeting daily step count recommendation 11,500 SC/day for preschoolers and 13,000/11000 SC/day for school-aged boys/girls											
	Families with non-overweight children						Families with overweight/obese children					
	%^a^	OR	95% CI	%^a^	OR	95% CI	%^a^	OR	95% CI	%^a^	OR	95% CI
Parent	Mother in the model (*n* = 582)			Father in the model (*n* = 357)			Mother in the model (*n* = 125)			Father in the model (*n* = 98)		
Step counts												
< 10,000 steps/day	38.7	Ref.		43.8	Ref.		27.6	Ref.		33.3	Ref.	
≥ 10,000 steps/day	67.1	3.50***	2.29–5.34	68.0	2.41**	1.37–4.26	47.6	2.98*	1.16–7.65	57.8	1.29	0.42–3.91
Screen time												
< 2 h per day	53.2	Ref.		59.2	Ref.		45.8	Ref.		48.1	Ref.	
≥ 2 h per day	50.8	0.98	0.59–1.65	52.7	0.99	0.56–1.78	15.8	0.14**	0.03–0.59	37.5	0.38	0.10–1.34
Weight statu*s*												
Normal weight	52.9	Ref.		65.1	Ref.		32.9	Ref.		58.1	Ref.	
Overweight/obesity	49.2	1.02	0.62–1.66	50.0	0.50*	0.28–0.89	35.5	1.09	0.43–2.77	37.0	0.42	0.13–1.34
Organized PA												
No	49.7	Ref.		54.9	Ref.		35.8	Ref.		51.6	Ref.	
Yes (≥1× per week)	56.0	1.10	0.71–1.71	56.1	0.62	0.34–1.12	34.1	0.53	0.17–1.62	39.3	0.46	0.13–1.65
Children												
Gender												
Boys	48.2	Ref.		52.3	Ref.		29.8	Ref.		35.6	Ref.	
Girls	54.2	1.16	0.77–1.77	57.8	0.86	0.50–1.50	41.9	3.17*	1.22–8.26	53.1	3.82*	1.05–13.91
School grade												
Preschool	55.1	Ref.		61.1	Ref.		42.9	Ref.		66.7	Ref.	
1st-3rd grade	48.4	0.80	0.46–1.41	52.7	0.60	0.29–1.24	33.8	0.23	0.05–1.16	38.3	0.31	0.06–1.68
4th–5th grade	51.7	0.95	0.58–1.56	53.4	0.72	0.38–1.37	33.1	0.32	0.07–1.42	41.3	0.70	0.13–3.72
Entertainment ST												
Non-excessive	55.2	Ref.		58.4	Ref.		44.3	Ref.		47.7	Ref.	
Excessive	46.6	0.75	0.45–1.23	48.6	0.55	0.29–1.05	24.4	0.29	0.07–1.17	37.9	0.80	0.22–2.85
Organized PA												
No	41.8	Ref.		43.4	Ref.		27.9	Ref.		45.2	Ref.	
Yes (≥1× per week)	57.6	1.80*	1.13–2.87	61.5	2.85***	1.54–5.27	41.7	2.37	0.88–6.40	50.0	1.99	0.64–6.22
Nagelkerke R^2^		0.15***			0.17***			0.37***			0.27***	

The models were adjusted for age category and gender of children

Organized PA – structured/organized leisure-time physical activity (e.g. sports training) does not include lessons of physical education during school/kindergarten time

Entertainment ST – sitting/lying while watching TV and sitting/lying in front of a PC (notebook, tablet, or smartphone) and not for school/work purposes (Excessive – more than 1 h/day for preschool children and more than 2 h/day for school-aged children; Non-excessive – less than the excessive amount)

%^a^ proportion of children (daughters, sons) who met the pedometer-based recommendation for daily step counts (a value of 11,500 steps/day for preschool children and a value of 13,000 steps/day for school-aged boys and 11,000 steps/day for school-aged girls) in the given area

*OR* odds ratio, *95% CI* confidence interval, *Ref.* reference group, *R*^*2*^ Nagelkerke coefficient of determination, logistic model, Enter method

The statistical significance is expressed as *p < 0.05, **p < 0.01, ***p < 0.001

While achievement of the recommended step count level by fathers significantly increased the odds of non-overweight children achieving the daily step count recommendations, parental overweight/obesity status significantly reduced these odds. The active participation of parents in organized leisure-time PA, regardless of their gender, did not significantly affect the odds of their children achieving the daily step count recommendations, regardless of their level of body weight. Conversely, non-overweight children participating in organized leisure-time PA at least once weekly were more likely to meet the recommended daily step count levels than their counterparts without organized leisure-time PA.

The active participation of children in organized leisure-time PA (at least once a week) was positively associated with a significantly higher daily step count on weekdays in non-overweight and overweight/obese boys in comparison with non-participants in such activities (Fig. 1). The significant difference in the daily step count between boys (girls) who participated in organized leisure-time PA and those who did not do so ranged from 1764 to 2152 (1408–1471) steps on weekdays. Except for non-overweight boys, no significant differences in the daily step count at weekends were found in terms of gender and body weight between participants and non-participants in organized leisure-time PA. For all children, regardless of gender, body weight, or participation in organized PA, a lower daily step count was visible at weekends than on school days (Fig. 1).

## Discussion

The plethora of studies confirm the influential role of parents on the PA of their children through a variety of mechanisms, including parents taking responsibility for PA care [33], support [8, 35, 36], encouragement [7], and engagement [33]. Nonetheless, it has not yet been explained sufficiently which of the parental health-related indicators help children achieve the recommended level of PA or how these indicators vary between non-overweight and overweight/obese children. The results of this three-cohort study extend the current knowledge in the area of the parent-child PA relationship in a random sample of families with non-overweight and overweight/obese preschool and school-aged children. Another original feature of the present study is represented by the gender- and age category-stratified analyses of daily step count (specifically, the achievement of the daily step count recommendations) in all the members of families with non-overweight and overweight/obese children.

In response to the specific objective of the study, it was revealed that maternal achievement of PA recommendation (≥ 10,000 steps/day) significantly helped all children, regardless of their body weight, to reach the recommended daily step count. And furthermore, the active participation of children in organized leisure-time PA increased the odds of all children achieving the daily step count recommendations; however, for overweight/obese children this remained non-significant. Many studies confirmed that there is a positive relationship between the objectively monitored PA (or proxy-reported screen time) of parents and their children [10, 17, 22–24, 26, 28, 37, 39, 74, 75]. However, only a few of them focused on the analyses of the parent-child relationship in terms of meeting the PA/screen time recommendations (daily hours of screen time [10, 75, 76]; pedometer-determined daily step count [39]; minutes spent daily on moderate-to-vigorous accelerometer-based PA [17]), or categorizing the level of PA/screen time (median of moderate-to-vigorous accelerometer-based PA [24]; median of Caltrac accelerometer counts per hour [28]; tertile of pedometer-determined daily step count [37]). The present study therefore adds to the body of knowledge regarding the existence of a relationship between parents’ and children’s achievement of the PA recommendations.

Similarly to other studies [22, 23, 26, 34], we found stronger positive relationships between mother-child PA than father-child PA in families with both non-overweight and overweight/obese children. In addition to those studies, we found that in families with overweight/obese children the mother’s behaviour (PA and screen time) is even more closely associated with their children’s PA than that of the father. On the other hand, only fathers’ body weight had a negative effect on the odds of their children meeting the step count recommendations. No such association was observed in our study regarding mothers’ body weight. Perhaps overweight/obese children are more likely to adopt patterns of parental behaviour than children of normal body weight. This idea is supported by the finding of lower differences in the daily step count among individual members of families with overweight/obese children compared to families with non-overweight children, where the child’s PA exceeds the parental activity.

The active participation of parents in organized leisure-time PA did not affect the odds of overweight/obese or non-overweight children achieving the daily step count recommendation. In one of the rare studies on the topic, Erkelenz et al. [77] also analysed the relationship of parental PA and the participation of their six-to-eight-year-old children in organized sports in addition to parent-child PA. They did not observe relationship between parental and children’s PA; however, children with at least one active parent displayed more minutes of participation in organized sports. The absence of a parent-child PA relationship but a higher level of participation of children in organized sport in the event of there being at least one more active parent indicates that parental support for children’s PA could be more important than parents’ joint PA [77]. The greater significance of parental support for children’s PA than of parent-child PA is also highlighted by meta-analytical studies [35, 36], which also encourage further verification of the relationship between parent-child PA and various types of parental support for their children’s PA and their children’s actual PA.

The present study showed that an active participation in organized leisure-time PA (at least once a week) is the only one of the anthropometric and behavioural correlates of non-overweight children that was analysed which significantly increased the odds of daily step count recommendations being met. In the case of overweight/obese children, active participation in organized leisure-time PA doubled the chance of their reaching the daily step count recommendation compared to those not attending organized leisure-time PA, but the result was not significant. Previous studies documented the positive contribution to the all-day objectively monitored PA of active participation in physical education lessons (step count, moderate-to-vigorous PA) in boys of normal weight and overweight/obese girls aged 9–11 [78, 79]. In addition, these studies reported a significantly higher proportion of boys of normal weight and overweight/obese girls who met the recommendation of 60 min of moderate-to-vigorous PA per day on a day with active participation in a physical education lesson than on a day without a physical education lesson. Therefore, it was expected that active participation in organized leisure-time PA would increase the chance of both non-overweight and overweight/obese children achieving the daily step count recommendations. However, a study among Finnish preschoolers did not reveal differences in light PA or moderate-or-vigorous PA among participants and non-participants in organized PA or between children of normal weight and overweight/obese children [23]. Significant differences in daily PA between participants and non-participants in organized leisure-time PA and between children of normal weight and overweight/obese children, as well as differences in PA between school days and weekend days, appear to be evident only after children start attending primary school [26, 27, 53].

We concur with the idea that parental correlates relating to PA and the overweight/obesity of their children are related in different ways in developing versus developed countries [17], and there is still a need for more detailed disclosure of parent-child PA relationships. The challenge lies in determining ways to effectively motivate and support parents and other caregivers of young children to optimize practices related to young children’s health-related behaviours [80]. There are several parental correlates of the objectively measured PA of preschool and school-aged children from Central and Eastern Europe that should receive more attention. It is necessary to include socioeconomic status of families and the level of parental education [7, 20, 23, 30, 81], incompleteness of families [6, 21], PA and support from classmates and siblings [7, 8], and the type of residence and quality of the neighbourhood [21].

### Strengths and limitations

The main strength of this three-cohort parent-child study is the involvement of all family members with preschool and school-aged children in the simultaneous monitoring of week-long ambulatory PA and screen time, as proxy-reported by parents. Moreover, contrary to comparable international studies [36, 80], the present research used stricter inclusion criteria for the final data analysis. Only data from children and their parents whose PA and screen time were monitored continuously for at least 8 h a day on at least four weekdays and both weekend days were analyses. This strictness provides a more valid comparison of the duration of parents’ and children’s daily step count and screen time between weekdays and weekend days, and helps reveal the variables increasing the odds of overweight/obesity among four-to-12-year-old Czech children. Another strength of the study is that the total amount of daily PA is supplemented with information about participation in organized sport.

However, the conclusions of any study need to be formulated under the spotlight of existing methodological limitations. Firstly, although the parental proxy-reported variables of their children’s health-related behaviour (PA and screen time) are considered to be valid and reliable for assessing PA and screen time levels, there is always a possible bias caused by social desirability. However, the parents, kindergarten/school teachers, and children were not told what age and gender-related step count and screen time recommendations exist prior to the commencement of the eight-day monitoring of PA or during it. Moreover, the data from the first day of measurement was also excluded from the final data analysis because the recording of the first day was incomplete and the novelty of wearing the Yamax pedometer might have affected the initial activity (reactivity) [51]. Second, the PACPAC study uses pedometers to objectively capture PA, but unlike accelerometers, these pendulum arm tools are not designed to collect information on bouts, type or, in particular, the intensity of PA. However, these less expensive devices are recommended as an inexpensive, small-sized, easy-to-use, and objective (valid, reliable, and non-reactive) method that provides a summary output of daylong ambulatory PA (quantified as the step count) of preschool [82, 83] and school-aged children [84] and adults [67] for the categorization of their achievement of the step count recommendations. Third, large differences in sample size and response rate of families with preschool and school-aged children could have a potential impact on the results of PA/screen time results. Families with preschool children were more likely to meet the inclusion criteria for weekly PA/screen time monitoring and provide valid anthropometric, PA/screen time data than families with school-aged children. It may seem that attitude of families with preschool children to research is more responsible compared to families with school children; but in families with school-aged children, a lower response rate could imply more time spent on school duties and the start of childhood puberty. Finally, the cross-sectional design of this study does not allow the causality of the parent-child PA relationships to be ascertained, despite their statistical significance. However, given the age of preschoolers and school-aged children, it is more likely that parent health-related behaviour affects the behaviour of children than vice versa.

## Conclusions

Altogether, the results of this study underline the differences in the parent-child PA relationship between families with non-overweight and overweight/obese children and highlight the effect of maternal health-related behaviour on their children’s PA. The mother’s achievement of PA recommendation (≥ 10,000 steps/day) significantly helps all children, regardless of their body weight, to reach the recommended daily step count. Conversely, excessive screen time (≥ 2 h per day) in the mothers of overweight/obese children significantly reduces the odds of their achieving the recommended daily step count. Whilst the active participation of parents in organized leisure-time PA is not related to their children’s PA, the active participation of children in organized leisure-time PA almost doubles or even multiplies the odds of their meeting the daily step count recommendation in both non-overweight and overweight/obese children. Involving all family members in inexpensive PA enhancement programmes (especially at weekends) and increasing the participation of all boys and girls, regardless of body weight and age category, in organized leisure-time PA could be a promising part of strategies for increasing daily PA and shaping an active lifestyle.

## Abbreviations

BMI: Body Mass Index
CI: Confidence interval
ICC: Intra class correlation
LR: Logistic regression
OR: Odds ratio
PA: Physical activity
PC: Computer use
Ref.: Reference group
SC: Step count
SD: Standard deviation
SPSS: Statistical package for the social sciences
ST: Screen time
TV: Television viewing
WHO: World Health Organization
